# A Miseducation: Perspectives on Sexuality Education from Black Women in the US South

**DOI:** 10.3390/ijerph21111516

**Published:** 2024-11-14

**Authors:** Rebecca Hailu Astatke, Yves-Yvette Evans, Stephanie Baker, Monica Simpson, Terri-Ann Thompson

**Affiliations:** 1 Department of Epidemiology, Harvard T.H. Chan School of Public Health, Boston, MA 02115, USA; rastatke@g.harvard.edu; 2 Ibis Reproductive Health, Cambridge, MA 02140, USA; yevans@wi.edu; 3 Department of Public Health Studies, Elon University, Elon, NC 27244, USA; sbaker18@elon.edu; 4 SisterSong Women of Color Reproductive Justice Collective, Atlanta, GA 30377, USA; monica@sistersong.net

**Keywords:** sexuality education, Black women, United States, community-engaged, qualitative, community-based participatory research, sexual and reproductive health, social barriers, racial inequity

## Abstract

Over the last three decades, the receipt of formal sexuality education has declined, with half of adolescents nationwide receiving the minimum Healthy People standard of sexuality education from 2015 to 2019. Further, racial and geographic inequities in sexuality education remain, with Black women and girls more likely to receive abstinence-only-until-marriage instruction. We sought to describe Black women’s sexual education in two southern states, North Carolina and Georgia. We conducted a qualitative community-based participatory research study. We held focus-group discussions with forty-nine Black women in Georgia and North Carolina between May 2019 and January 2020. The research team, the reproductive justice organization, and the Research Board reviewed, discussed, and refined themes developed using deductive thematic analysis. Most participants were employed. The median age was twenty-seven. From the participants’ accounts, we observed the inadequacy of sexuality education and the resulting process of unlearning inaccurate, negative information and learning positive and accurate information about sexuality. Participants expressed a desire for accessible, high-quality sexual education for themselves and the next generation that addresses autonomy, pleasure, and consent. Our findings highlight the need for investment in existing community efforts and in creating high-quality, culturally responsive comprehensive sexuality education nationwide to effectively address structural barriers to accessing sexuality and relationship information and skills.

## 1. Introduction

Receipt of formal sexuality education has declined in the US over the last three decades, with only about half of adolescents from 2015 to 2019 receiving sexuality education that meets the minimum standard articulated by the Healthy People 2030 guidelines [1,2,3,4,5]. Factors contributing to this decline include a lack of federal programs dedicated to funding and expanding access to comprehensive sexuality education (CSE) and the refusal of many states to accept sexuality education funding [1,2,3,4], out of concern for topics like contraception and queer theory being included in curricula and abstinence-only mandates [6,7].

High-quality CSE, as defined by the Sexuality Information and Education Council of the United States (SIECUS), provides culturally suitable, age-and-stage-appropriate, science-based, medically accurate information and skills regarding sexuality in schools from Grades K through 12 [8,9]. Research shows that CSE effectively reduces rates of unintended pregnancy and sexually transmitted infections (STIs); increases earlier use of more effective forms of contraception; improves communication with partners and parents regarding sex and reproductive health topics; and enhances students’ understanding of bodily autonomy [10], among other important impacts [2,9,11]. In contrast, abstinence-only-until-marriage (AOUM) programs result in poor short- and long-term reproductive and sexual health outcomes and higher community health burdens [12,13,14].

The majority of AOUM programs take place in the South [15]. Only six of the sixteen states and the District of Columbia (DC) that make up the US South mandate medically accurate content [16,17], while fourteen stress abstinence and require parental involvement [16]. Only three states require that consent [10] be taught, and none provide comprehensive healthy relationship instruction that addresses consent, violence prevention, communication, and decision-making skills [16,17]. Moreover, four states provide sexuality education that includes discriminatory information on sexual orientation and gender identity; fifteen states and DC permit the promotion of religion and do not mandate culturally appropriate and unbiased sexuality education [6,17]. This concentration of AOUM instruction in the South has pronounced consequences, perpetuating structural barriers for Black women, as 59% of the nation’s Black population resides in this region, and this hinders continued efforts by community organizations to improve the quality of and access to sexuality education for this demographic [2,9,18].

Existing research on Black women’s reproductive health has focused on contrasting differences between Black women and other racial/ethnic groups’ reproductive health outcomes. And while it is acknowledged that the lived experience of Black women contributes to these differences, few studies examine the mechanisms and complex factors by which this lived experience influences perspectives of and processes related to reproductive health [15,16]. Attempts to improve Black women’s sexual and reproductive health outcomes by changing their sexual knowledge have used theoretical frameworks such as the health belief model [19] and centered health promotion interventions—but the content and interventions have not been developed or informed by the community, limiting their utility [20]. Community-based participatory research (CBPR) generates informed, community-specific data that can be applied to create more sustainable interventions [21], effective dissemination and translation of findings [22,23] and, in turn, better health outcomes [24,25].

Prior research has shown that AOUM programming in schools and its negative effects disproportionately impacts Black youth, with greater implications for those residing in the US south, where AOUM prevails [26]. Regarding Black women’s perspectives on sexuality education, very little research has been conducted [12,27,28]. One study of forty-eight African American young people in Michigan reported that AOUM programming was limited and that young people desired information on health, pleasure [29,30,31], and emotional and relational aspects of sexuality in addition to abstinence [27]. Participants relied on solicited and unsolicited information outside of the classroom to fill the gap [27]. Ultimately, the study found that participants wanted more relevant, accessible, youth-centered comprehensive sex education that emphasized self-determination, trust, and credibility as core principles [27]. Two other studies were conducted among individuals residing in rural communities in North Carolina [12,28]. The first examined the role of public schools in HIV prevention among ninety-three African American youths and identified AOUM programming policies and practices as barriers. In this study, participants called on schools to provide access to health services and comprehensive sex education [28]. The other study included twelve African American adolescents and twelve adult community actors, including parents, religious figures, and afterschool program directors [12]. The main findings suggested that participants found comprehensive sex education acceptable; thought sexuality education should be “all-inclusive” and incorporate values and appropriate timing of sex; and believed parents and schools were responsible for providing youth with this information [12].

The Trust Black Women (TBW) partnership, a coalition of Black leaders and organizations that mobilized to defend against campaigns and legislation in Georgia that threatened reproductive justice for Black women in 2010 [32], collaborated with a research organization to conduct a study on Black women’s sexual and reproductive experiences across the life course. The study expands on the existing body of sexuality education literature by adding narratives from a new southern state and by offering an intentionally generational, intersectional, and community-oriented understanding of sexuality education for and by Black women. Further, it uses a qualitative and CBPR approach to amplify the voice and experience of Black women and generate meaningful recommendations for programming and policy based on what was important to them. In this paper, we present findings from our analysis of Black women’s experiences with sexuality education in two Southern states, North Carolina and Georgia. Based on these data, we aimed to understand how Black women processed the sexuality education they received throughout their lives.

## 2. Materials and Methods

### 2.1. Positionality

The study team consisted of Black women who received sex education across the south and other parts of the United States. They represent a range of educational backgrounds, generational experiences, reproductive health histories, and sexual orientations.

### 2.2. Study Data

The study team used a qualitative CBPR design to integrate community and research perspectives into every phase of the research study, from design through to analysis and dissemination. Study team members belonging to a reproductive justice organization, two community organizations affiliated with the work of the TBW partnership, and a Research Board of Black women who resided in Georgia or North Carolina and engaged with sexual and reproductive health work represented community perspectives. Two Black female researchers with knowledge and training in qualitative methods and community-engaged frameworks at a nonprofit research institution represented the research perspectives. Additional details about the CBPR process can be found in the work of Thompson et al., 2022 [33].

Eligible participants were 18–49 years old, English-speaking, self-identified as Black or African American, and had lived in Georgia or North Carolina for at least two years. Leaders from the two community organizations led recruitment and data collection. Recruitment efforts focused on urban and suburban centers and used a variety of methods, such as email lists, social media, and flyers posted at health clinics or other spaces where Black women could be reached.

**Figure 1:**
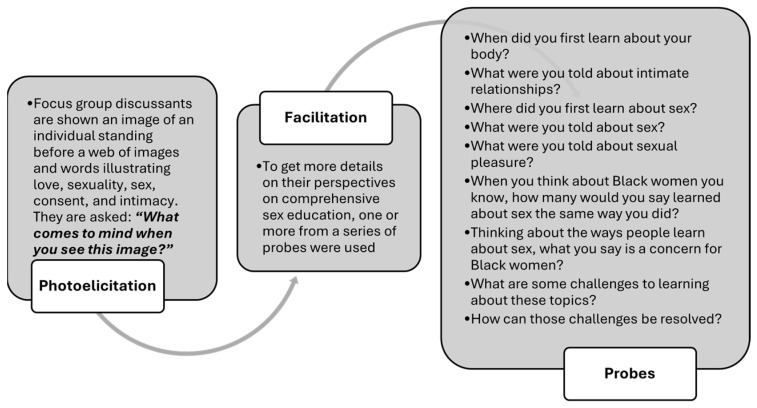
Focus group facilitation process for sexuality education.

The study team developed topics and probes for the focus group discussion (FGD) guide based on a review of sexual and reproductive health literature. A photo-elicitation method was used to evoke narratives around each FGD topic in the guide. Participants were shown an image related to the topic of sexuality education and asked to speak on what came to mind. Probes were used to elicit greater detail on the narratives raised. An illustration of the process used in addition to a sample of the probes is captured in Figure 1. Based on findings from the few existing CBPR qualitative studies among Black people that demonstrate the failings of AOUM programming and a desire for high-quality CSE with open communication on diverse content [12,27,28], we hypothesized that our participants would describe similar experiences with AOUM programming and its limitations, as well as a preference for CSE.

Researchers on the study team trained community organizations in FGD facilitation at the start of the study. Between May 2019 and January 2020, community partners of the same sex and race as the 49 women FGD participants conducted a total of 6 FGDs in the community organizations’ offices, 4 in Georgia and 2 in North Carolina, with 8–12 participants each. To obtain diverse responses, FGDs were conducted with different age groups: ages 18–49 (3), 25–49 (1), and 18–24 (2). On average, FGDs lasted 122 min. The same semi-structured FGD guide was used in both states. We used an audio transcription service to transcribe narratives verbatim.

Participants received USD 50 for taking part in the study. Ethical approval was received from an independent Institutional Review Board, the Allendale Investigational Review Board. All study participants provided informed consent.

### 2.3. Analysis

Using the guides as a framework, the research team developed codebooks for all FGDs. Authors (TT, YY) double coded each FGD, reconciled differences between the codes, discussed new codes and/or sub-codes, and applied a set of codes to all subsequent transcripts based on consensus. All codes were presented to, discussed, and categorized with the community organization, reproductive justice organization, and Board. For this paper, we include four categories of codes specific to sexuality education and sexuality: body lessons; sexuality lessons; providing sexuality education; and reproductive health education experiences. There were no sexuality education codes specific to race, gender, and age, although these elements of participants’ identities were present throughout discussions. The research team (RA, TT) used a deductive thematic analysis to identify themes across the four categories with the qualitative software Dedoose [34,35]. Members of the research team (RA, TT, YY), the reproductive justice organization (MS), and Board (SB) reviewed, discussed, refined, and finalized the themes.

## 3. Results

### 3.1. Participant Characteristics

Participants ranged in age from eighteen to forty-seven. The educational level was evenly distributed. Most of the participants were employed and fifty-five percent reported that they had been pregnant (see Table 1). The spread of participants’ educational and reproductive health history in each FGD resulted in cross-generational and intersectional discussions.

### 3.2. Findings

Several themes emerged from our analyses of what participants ultimately described as sexuality miseducation. Namely, their sexuality education was imbued with shame and stigma and healthy relationship education was absent. Participants underwent the burdensome process of unlearning this miseducation and learning accurate and diversified information. Additionally, many actively disrupted the cycle of miseducation to guarantee sexual well-being for the next generation. Quotes are identified by state and FGD age range.

*Theme 1*. Formative Information About Bodies and Sex Was Available from Multiple Sources but Emphasized Shame and Stigma.

Consistent with current sexuality education literature, most participants learned about the body, sexuality, and relationships from multiple information sources. These most often included families and schools, as well as churches, friends, peers, community, the internet, music, and media.

Participants described formative experiences of unwanted, demeaning, objectifying, and negative attention regarding their bodies from family and community members, especially around the onset of puberty. Participants understood this monitoring and disciplining of their bodies as “othering” principally due to their race, age, and gender. For some, othering led to an embodied shame and resulting disconnection with their changing bodies, sexuality, and relationships.

*“So, I would say Black girls tend to be sexualized at a young age. I remember wanting to dance […] I wasn’t trying to be sexual […] I learned from a young age that people saw me in a sexual light, […] it just always felt like it was a lot of shame that I didn’t have any control over. So, it was very hard for me to identify with myself sexually in a way where I wasn’t feeling like I was sinning. It was just a lot of shame in that period, and guilt.”*

*(NC, 18–49)*

Conversations with families and schools about sex were primarily described as avoidant and centering caution. Caution is a communication strategy that is well documented in sexuality education literature to prevent sexual abuse, unplanned pregnancy, STIs, and other consequential experiences, such as disruption of life plans and financial stress.

*“ […] I do believe in the African American culture, we all have heard when someone is saying “don’t do this”, and you’d be like, “why?” “Because I said so.” That’s all well and good. But, […] You have to be honest, you have to be willing to create that space for those questions to happen and just talk about it.”*

*(NC, 18–49)*

In addition to the use of cautionary communication strategies, the delivery of sexuality-based information, commonly based in fear and shame, discouraged participants from engaging in or furthering conversations with family members or educators. However, participants wanted open dialog and spaces that felt comfortable to have these conversations.

*“Not so much of what I wish they told me […] I think it would be more of an open space to be comfortable to ask those different questions for clarity. A lot of times when we go into the classrooms you’re getting told this, this, and this and okay that’s it, cool. You’re not comfortable enough or you don’t have that space to say, okay, so what is, where does, or how does, things like that.” *

*(NC, 18–49)*

*Theme 2*. Experiences of Sexuality Education Were Narrow and Lacking in Skills Key to Healthy Relationship Formation.

Discussions typically framed sex as a risky behavior or one engaged in for the sole purpose of reproduction and focused on preventing unplanned pregnancy and STIs. Timely, accurate information on a wide range of topics, such as puberty, pleasure, desire, intimacy, sex, love, emotions, relationships, and communication, was absent. Positive, informative experiences with sexuality education appeared to be exceptional instances dependent on interventions by individuals. For example, a participant at an AOUM school described a teacher who taught about safe sex practices:

*“At the middle school I went to, […] they pulled all the girls aside and had them make vows of chastity. And I have to say, I was so thankful for my health teacher because, […] most likely, a lot of the stuff that she did in that classroom, she was not supposed to do. I would bet money that she was not supposed to teach us how to use a condom and show us on a banana how you put a condom on and teach all the things that she taught us, but she did anyway.” *

*(GA, 25–49)*

Many noted that healthy relationship education that included conversations about developing values and expectations in relationships, cultivating intimacy through emotional expression and knowledge, and consent was unavailable, despite being essential to relationship development founded in trust, care, safety, and reciprocity. Without health relationship education, participants relied on media and their interpersonal experiences to inform their understanding, resulting in unrealistic or singular examples. Accordingly, some participants reported experiencing difficulties with communication (negotiation and reconciliation skills); decision-making (i.e., developing values and expectations in relationships); and social and emotional learning (i.e., cultivating intimacy through emotional expression and knowledge).

*“The way that I learned about most of the reproductive stuff—I was in school in Georgia. […] But the other stuff, like all of that heavy stuff and emotion stuff and consent stuff and all those things, I didn’t have any of those tools, and nobody gave me any of those tools, and I’m 34 years old now and I’m still trying to navigate that stuff.”*

*(GA, 25–49)*

*“And not that I want to take responsibility away from people who did me wrong […] However, I also take responsibility for myself because I know that there was something I didn’t have. I didn’t have the tools for that. I didn’t know how to navigate that situation. I didn’t understand my worth. I didn’t know how to set expectations in a relationship. I didn’t understand basic emotional requirements of a relationship. I just didn’t know—I didn’t know how to do that […] because nobody told me what my rights were. Nobody explained to me that I really only have to be responsible to myself—that I don’t owe anybody anything, and that nobody owes me anything.”*

*(GA, 25–49)*

*Theme 3*. Inadequate and Inaccurate Sexuality Education Created an Ongoing Process of Learning and Unlearning.

The confluence of limited, absent, and inaccurate sexuality education content spanned multiple generations and set in motion a process of learning and unlearning. As two participants described:

*“From the Black women that I’m around now, we’re still figuring it out or realizing we didn’t have it figured out. Especially me and my friend group now, it’s like what we thought was sex really isn’t sex and what we thought was normal—even just relationships and we talked about all of that stuff … even aunts and cousins who are in their 40s, 50s, like they are still figuring it out.”*

*(GA, 18–24)*

*“I’m 47 and I’m still trying to navigate and really unlearn some of the things that I learned that were absolutely false.”*

*(GA, 25–49)*

The process of learning often started with a recognition of a gap in knowledge and was ongoing. For some, the recognition of limited sexual knowledge and the work to fill those gaps started early but for others, that recognition did not surface until adulthood. To fill knowledge gaps, participants described seeking out information on sexuality. Others described learning about sexuality as they got older through peer mentoring, birthwork, and sex-related activities like erotic labor and stripping. For instance, one participant mentioned choosing friends whose parents talked to their kids about sex when they were younger. One participant’s journey in learning about menstruation, ovulation, and pregnancy illustrates how some fill knowledge gaps and the span of time it can take to acquire comprehensive knowledge on a specific reproductive health topic in the absence of quality sexuality education.

*“When I was like 9 or 10, I checked out this book from the library. […], and that’s really when I learned about like menstrual periods and how pregnancy happens. And that was like the knowledge I rolled with until I was about like 20-something when I really learned more about the clitoris and this and that […], so basically I was a grown adult before I really, really knew how sex happened. And even the more specific stuff about pregnancy, like I kind of knew the basics, but I didn’t know like oh, you really mainly get pregnant during ovulation, […]. I didn’t really know that until I was maybe like 25.”*

*(GA, 18–49)*

Illustrations of the unlearning process were often framed through intra- and inter-personal experiences. In particular, participants unlearned relationships as an experience of forfeiting their autonomy, being compromised, being acted upon, and in service of others.

*“[…] I thought sex was something that happened to women. Like, most women don’t want to have sex, but you got a boyfriend or you got a husband. Sex is part of the package. It is what it is. It’s not really something for you. It’s something for them […]”*

*(NC, 18–24)*

They unlearned relationship roles and dynamics that discouraged expressing vulnerability, such as caretaking and co-dependence based in stereotypes of their identities as Black women.

*“… And just thinking about […] the way that my mom cared about people is like she will let people stay with us in our house because they’re in need or she’ll give her shirt off her back or borrow people money. And so, what I learned about intimate relationships is that you sacrifice yourself for them and that you take care of people and the reason why you’re with people is because they need you […]”*

*(GA, 18–49)*

Over time, participants realized their capacity to act as agents, to participate in their relational lives and to do so freely. They changed their perspectives on sexuality from being focused on the sexual act and sex as a risky behavior to a more holistic perspective—one that emphasized self-trust, autonomy, consent, pleasure, confidence, trust, care, ease, and joy as part of the sexual experience.

*“I got to college and I was like, Oh, […] This is actually how I’m supposed to experience this, and I didn’t know that. I also didn’t know that I have some agency in this. Actually, you know like, it’s kind of lit.”*

*(NC, 18–24)*

*“You always have the power to say no—like not just in sexual situations but in any aspect along the way. Consent shows up in so many different ways and […] because you are an autonomous person you always have the chance to say no, and […] at the end of the day—as long as you feel happy and secure in the spaces that you’re in, you don’t have to compromise that to be wanted by somebody else […]”*

*(GA, 18–24)*

*“I wish somebody would’ve told me pleasure is abundant. You can get it from a lot of different places. I was similar to that too, thinking I’m not going to find this nowhere else [….] I wanted to keep a firm grip on this thing that I found that I never had before, because I don’t think that I’m going to get it back. And that’s not true. It’s not true.”*

*(NC, 18–24)*

*“[…] It’s [sex] something that you only have with one person and you all just do it in this one position forever and that’s it. […] like as long as the one person, i.e., my husband, is happy. Good. That’s it, […] Now I look at sex as such a freeing thing […]”*

*(NC, 18–49)*

In addition to gaining greater self-trust, some participants described learning to center themselves, especially in relationships, by doing what was best for them with their own values and at their own pace. They learned how to express emotions, connect with others, and communicate more authentically.

*“…So, my view on relationships have to be based off of me now. I have to make that decision. I have to determine whether—hey. Is this person really fit, a good you know lifestyle for me to even put myself out there? And that’s how I am now.” *

*(GA, 18–49)*

*“[…] I’m not going to get pleasure out of this if we don’t talk about it. And so for me it’s just having that conversation […]”*

*(GA, 18–24)*

Moreover, centering themselves meant reconnecting with their bodies. The disconnection that resulted from the distress of unexpected changes, such as the common experience of menarche without any knowledge of menstruation, was replaced by an awareness and ensuing comfort with their bodies. They learned to accept their bodies as ever-changing by adopting a persevering attitude with assurance and trust in themselves.

*“But so it was really weird for me to—like this journey that I’ve been on with my body since I was 9 years old of really feeling connected and inhabiting my body, not just like something that feels so disconnected from my being and I think like this is probably the first time in my life, for real, that I’m just fully connected and feel at home in my body even though it’s changing because […] it’s just like the last few years have just been really weird, […]—got diagnosed with PCOS like three years ago, and so I’ve had to relearn my body and that’s been—it feels like every five years I have to learn something new about my body to become comfortable with it and then it’s like every time I get comfortable with it something else changes”*

*(GA, 18–24)*

*Theme 4*. Garnering Sexual Well-Being Meant Disrupting the Cycle of Miseducation for Themselves and the Next Generation.

Despite their inadequate sexuality education, participants engaged in a burdensome but liberatory process of unlearning and learning sexuality education to establish reciprocal, intimate, and joyful relationships with themselves and others. This experience prompted some to act to disrupt the cycle of misinformation for their children, their grandchildren, and the generations to come. One participant shared about her granddaughter:

*“[…] I don’t want this to be generational. I don’t want this to be her experience. […]. I want her to know her body. I want to talk about orgasms and tell her how exciting when she finds the right situation it’s going to be […], not look at sex as a bad thing. If sex is a bad thing, now why would you have me.”*

*(GA, 25–49)*

While many participants noted that societal perspectives on sexuality had changed for the better, with more discussions of body positivity, sex positivity, and acceptance of sexual and gender diversity, they worried that without standard sexuality education, individuals would be left with mixed and incomplete sexuality education, dependent on the unique family and community structures constituting one’s spheres of influence. This was deemed unacceptable for the next generation.

Disrupting the cycle of misinformation was not considered an easy task. Many found it daunting and expressed their trepidation, especially without any precedent.

*“[…] no one taught me anything. I worked at a library, I had to go look this stuff up myself, which is why, I promise you I have sons and I’m so glad because then there’s no way, how am I going to explain something to a daughter? I don’t even know. Like, I mean now I’m an adult, but there was no conversation. No conversation.”*

*(GA, 18–49)*

Even so, they took action, cultivating an education that went beyond simple information exchange and offered the empowering sexuality education they had desired. For many, this started with communication based in language that was clear and shame-free. They had and encouraged direct, positive, back-and-forth dialog.

*“With my son, I have a three-year-old, he has a penis, he doesn’t have a pee pee, […] we have to talk about it because if we’re not talking, it’s just going to keep going generation to generation.”*

*(NC, 18–49)*

*“Things that I wanted them [the next generation] to know is just be confident and comfortable with their body knowing that all the things that your body does, I think, is really important […]”*

*(GA, 18–49)*

Their efforts to foster open and comfortable conversations were based in lessons on autonomy, pleasure, and consent, and intimacy rather than shame, stigma, and othering. Ultimately, in connecting with themselves through lessons from their own lives, they connected with others as well. By recognizing their own intrinsic empowerment, they expanded and shared these embodied lessons of autonomy, empowerment, intimacy, communication, consent, and pleasure amidst pervasive disempowerment, shame, stigma, and othering. Two parents describe the process of paying it forward:

*“I’m very grateful because I have two daughters and a son whose awareness of their sexuality and who they are and how they are in their confidence is decades beyond where I was at 20 and 18 and 15. Like their confidence in who they are, their sexuality, their understanding. They ask me anything. And there’s been things they’ve asked me and they’ve shown me and told me, especially like the understanding with the generation now, talking about LGBTQIA and how this works and how that works.”*

*(GA, 25–49)*

*“And so, I talk to my kids now. I tell them everything. My children are 4 and 8 and they know everything. Any questions that they have, I answer all of them clearly, using anatomical charts. You are the master of your own body. I don’t even have a right to touch you in a way that you don’t want to be touched because that’s everything—because I was taught that I didn’t matter. I was taught that my feelings should be minimized and that I was for the pleasure and consumption of other people, and that was my pertinence, and that’s what I learned. And that’s what I’m still trying to unlearn.”*

*(GA, 25–49)*

## 4. Discussion

Our study contributes to the growing research on Black women’s perspectives and experiences of sexuality education and its impact on well-being. Participants highlighted inadequate education not only on foundational concepts such as safe sex, but also missing information on intimacy and relationship formation. They described continuously learning about their bodies, sex, and relationships and unlearning inaccurate sexual information, alongside common experiences of shame, stigma, and othering. Relationships, autonomy, pleasure, and consent were emphasized as key and reinforcing elements for creating a more sex-positive and autonomous generation. They desired culturally responsive CSE for themselves and the next generation, and many worked to make that a reality within their families.

Similarly to our findings, participants from two studies in rural North Carolina [12,28] and another study in urban Flint, Michigan [27] described current sexuality education as inadequate. They expressed the desire for sexuality education that was initiated early, with regular open communication [12,27,28]; hands-on discussions of practical skills (e.g., condom usage) [27]; and a greater focus on the emotional and relational aspects of sexuality [27,28]. Our study expands upon the literature by highlighting miseducation as generational in our participants’ lives. Additionally, we show that inadequate education led to the burdensome process of learning and unlearning sexuality information throughout the course of a lifetime. Our findings suggest that a pathway to achieve sexual well-being includes fostering expansive knowledge, discussion, and skills around bodily autonomy, consent, pleasure, and intimacy, such as with a pleasure-centered lens, more healthy relationship content, and greater inclusion of interactive, skills-based sexuality education.

Previous studies [12,27,28] corroborate our participants’ views on sources of information for sex education and communication strategies, emphasizing the challenges parents face in being the primary source of sexuality education and the impact of caution and avoidance as common communication strategies. Our participants noted not only how these strategies contributed to stigma and shame intergenerationally, but also how communication was informed by the qualities of relationships within different family structures and relationship qualities [36]. Although families are largely responsible for providing information and skills regarding sexuality education [10], interventions focused on improving parent–teenager communication about sex within Black families indicate that parents’ own need for sexuality education creates significant barriers in effective delivery [12,37,38]. Schools as a primary source of sexuality education have been viewed as the most effective for Black students [12,27,28] and offer ways to support families’ diverse experiences with sexuality education. However, school sexuality education needs to be strengthened with CSE curricula. These standards have been proven to improve communication with partners and parents on sex and reproductive health topics, including those topics which our participants noted as missing or misrepresented [2,11].

Moreover, sexuality education situated within a reproductive justice framework, where sexual development is a lifelong process of learning information and developing values [2], is needed to effectively dismantle tropes, historic injustices, and inequities in sexuality education [2,9,39,40]. Previous literature has focused on inequities within sexuality education, as well as reproductive healthcare more broadly. Findings from these studies have yet to be fully incorporated into CSE programming [27,33,39,41]. The lack of culturally congruent sexuality education can over-emphasize caution at the expense of strength-based education, often excluding topics like healthy relationships, consent, and pleasure [40,42]. Notably, these are the matters our participants raised as unequivocally valuable, alongside the influence of culture on the communication strategies employed, to fulfill sexual well-being.

Accordingly, a multilevel approach to CSE programming that is responsive to the shift towards a positive view of sexuality would be valuable [9,42]. High-quality CSE’s culturally responsive style of open dialog caters to intersectional and adaptive approaches best suited to the individuals present in the classroom [42]. It supports sexuality education that spans the life course, institutional settings, and cultural contexts [37,42]. High-quality CSE can interrupt the ongoing process of learning and unlearning our participants described. The benefit of multilevel programming is the coupling of accessible, sustainable improvements at the community level with those at the individual and interpersonal level, such as ameliorating low self-esteem and othering—especially racism and sexism—which our participants highlighted [39].

Our study had some limitations. Participants reflected on sexuality education over their lifetime. For some, recollections of formal sexuality education were within 7 years of adulthood (age 18), while for others, the period was much longer. Recall bias may be a concern, particularly for older participants. The focus on Georgia and North Carolina limits the generalizability of the findings to other southern states. Restricting our recruitment to urban and suburban areas means that perspectives from Black women living in rural areas are missing. (Two previous studies have focused on this population, specifically rural North Carolina [12,28].) Additionally, while the experiences of sexuality education described by participants were predominantly received in the US South, our narratives include some descriptions of sexuality education received in other states. We are missing demographic details for some participants, which may limit our understanding of how demographics influenced the narratives. Additionally, the perspectives of trans, nonbinary, and gender-expansive Black people are missing. Finally, while our findings were consistent across FGDs and resonated well with our community members, we were unable to validate these results through forums with participants.

Accompanying these, we note several strengths. Most importantly, our findings capture elements of sexuality education that are important to Black women, their desires for healthy relationships, and what they believe will make it possible. This study focuses on Black women in the south, the largest US regional area, who have a unique experience and whose voices deserve to be amplified. The use of a CBPR framework in conjunction with a study design that relied on photo-elicitation promoted rich and candid discussions led and raised by participants. Recommendations for sexuality education are often based in medical literature and limited to a set of reproductive health outcomes [9]. However, this is a small part of what participants express as constituting the desired elements of sexuality education. Namely, matters like healthy relationships, pleasure, consent, and sexual agency, all things that were absent in their education, had the most gravity in our participants’ sexual identities and well-being. Strengths-based CSE must recognize and mitigate the historical and ongoing practices that oppress, subjugate, and deny rights to Black women to be effective [39]. Our research findings show the value of incorporating Black women’s perspectives in practice and policies that affect their health.

### Policy Implications

The Department of Health and Human Services Action Plan to Reduce Racial and Ethnic Health Disparities and Healthy People 2030 have recommended broad strategies to reverse historical patterns of poor sexual and reproductive health outcomes among Black women [39,43]. Strategies include ensuring culturally and contextually appropriate research and prevention; equal access to effective sexual health information and quality healthcare services; quality education and training for public health professionals; and policies that promote sexual and reproductive health equity [39]. Importantly, most Black parents and students (90%) support CSE [2]. This sentiment is broadly shared, with steadfast support over the past two decades by 80–85% of parents nationwide, for school-based sexuality education that is comprehensive, medically accurate, and age-appropriate [11,44]. Despite this, policy at the federal, state, and local level continues to block CSE, leaving local organizations overburdened and public school systems unable to provide adequate instruction to the communities that are most affected [1,4,5]. For reasons such as AOUM mandates in curricula [6] or discriminatory curricula laws for sexual and gender minorities [7], many states, especially those in the US South, have not utilized funds. As a result, Black youth are left dependent on short-term and underfunded community-driven efforts. For instance, since 2013, many states have opted not to apply for grants to State Education Agencies (SEAs) and Large Municipal Education Agencies (LEAs) offered by the Centers for Disease Control and Prevention (CDC) to implement Exemplary Sexual Health Education (ESHE), an evidence-based approach that emphasizes continuous learning from K-12 [2]. Because many individuals have never received high-quality instruction throughout their lifetime, especially Black women who are no longer school-aged [45], it is largely community efforts that have worked to support these individuals through programming, workshops, or counseling. Our findings support the efforts of organizations like SIECUS who advocate for CSE that emphasizes a broad curriculum covering topics like consent, sexual orientation, gender identity, contraception, and STI prevention [8]. They also support organizations such as Kimbritive and Black Women Wellness that have begun to deploy cross-generational sexuality education programs [46,47]. Even so, the need for policy that supports sexuality education over the life course remains. Accordingly, we recommend passing the Youth Access to Sexual Health Services Act (YASHS) and Real Education for Healthy Youth Act (REHYA) [11,48]. YASHS would provide community grants to support programs and partnerships to bolster access to sexual and reproductive healthcare services for Black girls, as well as other young people facing barriers to sexual healthcare [11]. This would be coupled with REHYA, which would—among other provisions—eliminate funding streams for AOUM programming and redirect those funds to CSE programs that offer informative and inclusive curricula [2,11,48,49]. No single program nor policy will fully improve the state of sexuality education; however, these measures could feasibly and sustainably bolster sexual well-being and ameliorate historical sexuality education inequities in the US.

Every individual has the human right to CSE, as protected by international human rights treaties and recognized by global entities like the World Health Organization, UNESCO, UNAIDS and the United Nations Population Fund (UNFPA). This right to access high-quality CSE should not be dependent on geography [50,51]. To ensure all Black students have access to high-quality sexuality education across all states, CSE should be mandated as a minimum standard nationally [2,52]. This mandate would help to address the structural factors, namely issues of systemic racism and other forms of oppression that obstruct access to information and services, which result in these regional inequities. Otherwise, sexuality education perpetuates the very harms it seeks to eliminate in health policy and practice [33,39,42,53,54]. Moreover, mandating CSE nationwide would provide the cultural shift towards a more open and tolerant view of sexuality [9].

## 5. Conclusions

These findings support the case for CSE nationally. Our exploration of Black women’s experiences of sexuality education in two southern states shows the enduring impacts of misinformation inherent to AOUM programming. Policies that promote culturally and contextually congruent CSE supports the ongoing work our participants are doing to disrupt the cycle of miseducation for the next generation and sets Black women up for better sexual and reproductive health experiences and outcomes.

## Figures and Tables

**Table 1:** Participant characteristics (*n* = 49).

Characteristics	Number (49) *	Percent (%)
**State**		
Georgia	29	
North Carolina	20	
**Age**		
Median (range)	27 (18–47)	
**Education**		
High School, Associate’s degree, trade, other	7	35
Bachelor’s degree	20	41
Graduate school or higher	9	18
**Reproductive health history**		
Ever pregnant	27	55
Never pregnant	19	39

* All cells may not total 100% due to missing values.

## Data Availability

The data presented in this article are not readily available because participants did not grant permission for wider use of the data.
